# Size effect on the mineralogy and chemistry of *Mytilus trossulus* shells from the southern Baltic Sea: implications for environmental monitoring

**DOI:** 10.1007/s10661-017-5901-y

**Published:** 2017-03-30

**Authors:** Anna Piwoni-Piórewicz, Piotr Kukliński, Stanislav Strekopytov, Emma Humphreys-Williams, Jens Najorka, Anna Iglikowska

**Affiliations:** 1 Institute of Oceanology, Polish Academy of Sciences, Powstańców Warszawy 55, 81-712 Sopot, Poland; 2 Department of Life Sciences, Natural History Museum, Cromwell Road, London, SW7 5BD UK; 3 Imaging and Analysis Centre, Natural History Museum, Cromwell Road, London, SW7 5BD UK

**Keywords:** *Mytilus*, Mineralogy, Geochemistry, Monitoring, Shell size, ICP-MS

## Abstract

Mussels have the ability to control biomineral production and chemical composition, producing shells with a range of functions. In addition to biological control, the environment also seems to influence the process of biomineralization; thus, shells can be used as archives of ambient water parameters during the calcium carbonate deposition. Past and present environmental conditions are recorded in the shells in the form of various proxies including Mg/Ca or Sr/Ca ratios. For such proxies to be accurate and robust, the influence of biological effects including the size of studied organism must be examined and eliminated or minimized, so that the environmental signal can be efficiently extracted. This study considers mineralogy and elemental composition of shells representing four size classes of *Mytilus trossulus* from the Baltic Sea. Obtained results suggest that mineralogy and chemical composition change throughout the shell development due to most likely a combination of environmental and biological factors. The content of aragonite increases with increasing shell size, while the bulk concentrations of Na, Cd, Cu, U, V, Zn and Pb were found to decrease with increasing height of the shells. Therefore, using mussels for environmental monitoring requires analysis of individuals in the same size range.

## Introduction

Many marine organisms produce exoskeletons in the form of a shell with specific properties. They perform a range of functions from providing primary defence against predators to delivering protection from external environmental stressors resulting from water parameters (Lowenstam 1981; Lowenstam and Weiner 1989; Mann 2001). Although the constitution of mollusc shell is mainly biologically and genetically controlled (Watabe and Wilbur 1960; Addadi and Weiner 1992; Belcher et al. 1996), environmental factors (e.g. temperature, salinity, water chemistry) also affect its elemental and mineral composition (Dodd 1965; Lorens and Bender 1980; Bourgoin 1990; Pitts and Wallace 1994; Klein et al. 1996a, b). Thus, sequentially formed calcium carbonate layers of shell record the growth histories, metabolism and environmental conditions in which the organism calcified (Fuge et al. 1993; Klein et al. 1996a, b; Stecher et al. 1996; Swart and Grottoli 2003; Strasser et al. 2008). Furthermore, comparison between the compositions of recent and fossilized shells gives an opportunity to estimate natural (pre-civilization) levels of trace element concentrations and to biomonitor the evolution of ecological parameters (Bourgoin 1990; Puente et al. 1996; Carroll et al. 2009). Such a comparison is especially important when instrumental records of oceanographic and climatic parameters are not available.

Bivalves, especially of the genus *Mytilus*, have been employed for decades as bioindicators in the marine environment (Smith et al. 1979; Bourgoin 1990; Puente et al. 1996; Stecher et al. 1996; Putten et al. 2000; Richardson 2001; Markich et al. 2002; Andral et al. 2004). They occupy widely distributed habitats in the modern oceans, from coastal, often brackish waters to more pelagic environments, as well as being relatively common throughout the fossil record since the Cretaceous (Freitas et al. 2006). Bivalve molluscs, as filter-feeding organisms, are able to concentrate various contaminants from ambient water due to the bioaccumulation process. Components present at often undetectable levels in water can be detected in bivalves due to their high bioaccumulation ability (Zuykov et al. 2013). Many studies suggest that chemical compositions of growth-layered mollusc shells were related to external environmental characteristics. Magnesium to calcium (Mg/Ca) and strontium to calcium (Sr/Ca) ratios in calcite and aragonite biominerals have been proposed as proxies of seawater temperature (Elderfield and Ganssen 2000; Lear et al. 2002; Schöne et al. 2011). Non-biogenic incorporation of manganese (Mn^2+^) into calcite has been found to correlate with dissolved Mn^2+^ concentration in seawater (Franklin and Morse 1983). Thus, Mn/Ca could potentially provide a proxy for dissolved Mn^2+^, whereby reflect the redox processes, primary production (Putten et al. 2000) and associated phytoplankton blooms (Lazareth et al. 2003). Barium to calcium (Ba/Ca) ratio has been linked with the timing and magnitude of diatom blooms (Thébault et al. 2009) or, alternatively, has been suggested to be used as an estuary-specific indicator of salinity (Gillikin et al. 2006; Poulain et al. 2015). Sodium to calcium (Na/Ca) ratio has also been proposed as a salinity indicator; however, the association of Na^+^ with organic matter and its mobility in the crystalline phase of a shell must be considered (Dalbeck 2008). Furthermore, metals such as copper (Cu), cobalt (Co), zinc (Zn), lead (Pb), cadmium (Cd), nickel (Ni), iron (Fe) and vanadium (V) are detected during pollution monitoring (Protasowicki et al. 2008; Youssef et al. 2016).

A robust marine geochemical proxy should depend on a single oceanographic parameter. However, recent studies have increasingly indicated that the use of the mineral and elemental composition of marine bivalve shells as proxies is not a simple task and factors controlling shell variability are not well enough understood (Putten et al. 2000; Freitas et al. 2006; Gillikin et al. 2005a; Lorrain et al. 2005). Multiple parameters, including environmental conditions, e.g. temperature or salinity, and physiological parameters, such as size, growth rate or metabolic activity, can influence these proxies, thereby confounding their use. Consequently, each potential proxy needs to be rigorously studied.

In this study, we focused on the effect of the size of the organism on the calcium carbonate polymorph produced and on the incorporation of trace elements into the shell. *Mytilus trossulus* has been selected as a model organism and the Gulf of Gdansk in the Baltic Sea, as a model locality. Generally, within a superfamily of Bivalvia, shell mineralogy is rather constant. Bivalves lay down two forms of calcium carbonate in their shells: aragonite and calcite. Shells may be wholly aragonitic, or may contain both aragonite and calcite, in separate monomineralic layers (Kennedy et al. 1969). Different crystal lattice of polymorphs affect the physical properties of the shells and trace element incorporation into them. It is already known that active incorporation of minor and trace elements during shell formation varies with the growth of the organisms (Rosenberg 1980). Therefore, it is important to examine the relationship between mineralogy and elemental accumulation in shells taking into account the biological processes. The amount of Sr and Mg incorporated into the shells of bivalve *Mytilus* sp. decreases with age (Dodd 1965). A similar trend was observed for Pb incorporation into the structure of shell-forming minerals in the abalone *Haliotis* spp. (Hirao et al. 1994; Arai et al. 2003). Changes in Sr concentrations related to the ontogenetic stage of the organisms have also been observed in the shell of *Mya arenaria*, for which Sr/Ca are correlated positively with the age of the individuals (Palacios et al. 1994). Similarly, Schöne et al. (2011) found that Sr/Ca and Mg/Ca ratios in *Arctica islandica* shells increased with age. According to Dodd (1966), shell age also affects the precipitation of aragonite, which seems to be strongly correlated with shell size in *Mytilus edulis*. These results confirm that before biomonitoring can be done reliably, the extent to which the composition of shells depends on the size of the organism must be thoroughly studied.

Therefore, the aim of this study is an analysis of the behavour of the 16 elements important either physiologically or in environmental monitoring: Ca, Fe, Mg, Na, Sr, Ba, Mn, Cd, Co, Cu, Ni, Pb, uranium (U), V, yttrium (Y) and Zn. Finding the variability of these elements within four size classes of a single bimineralic species *M. trossulus* will likely increase the validity of using this species as a tool for environmental monitoring in the Baltic Sea and other regions within its extent of occurrence.

## Material and methods

### Study area

**Fig. 1:**
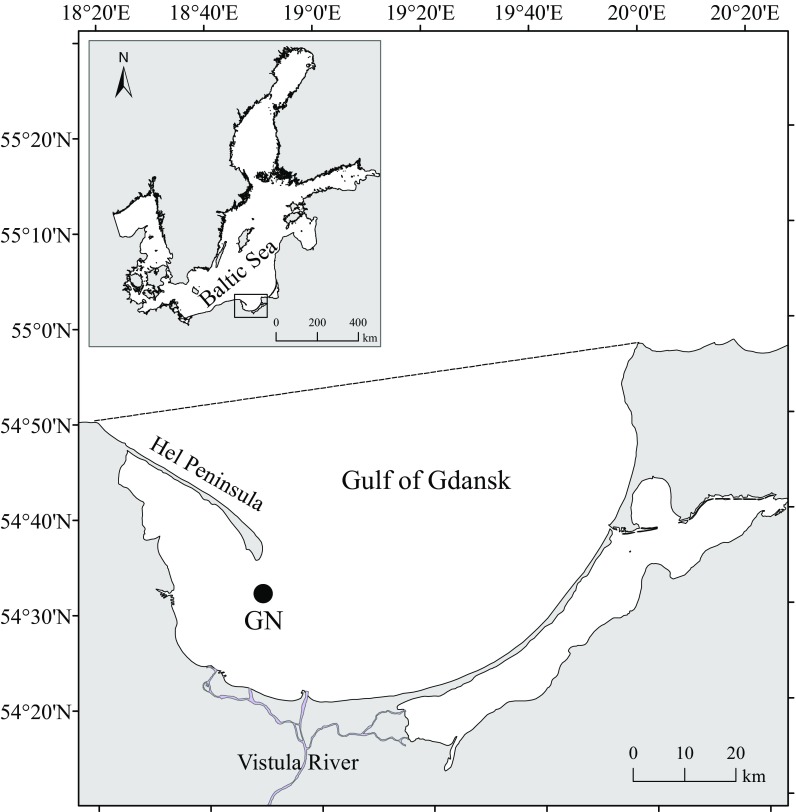
Study area and the site of collection (GN) of *M. trossulus* in the Gulf of Gdansk and its location in the Baltic Sea. The *broken line* indicates the northern border between the Gulf of Gdansk and the open sea

Samples were obtained from the Gulf of Gdansk located in the southern region of the Baltic Sea (Fig. 1). The gulf is open, but partially sheltered, being bordered to the north-west by the Hel Peninsula and to the west and south by Polish coastline (Rainbow et al. 2004). The location is under the influence of north-east winds, which can cause storm waves reaching a height of 4–5 m. The sea is the most turbulent in January, and the quietest in June. The wind, in conjunction with the bottom topography and shore morphology, is the main reason for the formation of currents in the Baltic Sea. In the Gulf of Gdansk, currents are created mainly due to the north-east exposition and the outflow of water from the west side of the bay, therefore dominating south-west and south-east currents with small average orbital speed near the bottom amounting 0.03 m s^−1^ with maximum up to 0.1 m s^−1^. Small tidal amplitudes, reaching a few centimetres, have only a minor importance for the induction of water currents (State Environmental Monitoring 2014).

An important feature of the southern Baltic Sea waters is seasonal variability of hydrophysical environmental parameters. The Baltic is characterized by three cyclic thermal systems: a summer system with the surface layer of warmest water (about 17–22 °C) of up to about 50 m, below which there is cold water from the previous winter; an isothermal system that forms in the autumn and spring, when in the whole water profile the temperature is almost equal (4–6 °C); and the winter system, when the surface temperature is the lowest (falls to −0.5 °C) and rises at the bottom to 4–6 °C (Uścinowicz 2011). The Gulf of Gdansk is a system of estuaries, where seasonal variability also affects the distribution of brackish water and seawater. An increase of melting, rains and river runoff during March and April leads to the salinity minimum. Then, the seawater inflow during autumn, the development of ice and vertical mixing in winter causes an increase in salinity. Consequently, the average salinity of waters in the Gulf of Gdansk varies from 5.5 in summer to 8.4 in winter (Bulnheim and Gosling 1988; Szefer et al. 2002).

Due to the costal location, the gulf is exposed to urban hazards. Variations in yearly temperature and salinity are minimally subjected to anthropogenic changes, which in turn, strongly modify the hydrochemical conditions of the reservoir. Emissions from coastal towns, ports, ships and atmosphere (Uścinowicz 2011) are discharged into the Gulf of Gdansk, making it a highly polluted sea area. The main freshwater inflow into the gulf comes from the Vistula River, which is the second largest input of river flowing into the Baltic Sea. The Vistula and its tributaries traverse highly industrialized and agricultural areas, featuring iron, steel, electrochemical, chemical, petroleum and refining industries, as well as mining industry (Szefer et al. 2002). HELCOM (2004) reported that the mean annual flow of Vistula is 1081 m^3^ s^−1^ and its plume might extend up to 9–27 km from the river mouth. According to the report of State Environmental Monitoring (2014), the Vistula River in 2011 was the source of 96.63 tons a year of Zn, 128.79 tons of Cu, 77.93 tons of Pb, 44.24 tons of Ni and 9.74 tons of Cd.

The cyclical nature of the environmental conditions in the gulf is closely linked with plankton blooms. The feeding behaviour of *M. trossulus* as a suspension feeder depends on the presence of particulate matter, mainly phytoplankton, reaching its biomass peak during spring and summer. During autumn and winter, phytoplankton growth is limited or stopped due to the decreased temperature, limited availability of light and even surface ice cover (Pierścieniak et al. 2010; Staniszewska et al. 2015).

The seasonality of temperature, salinity, water chemistry and changing dominance of plankton has a huge impact on the saturation state (Ω) of calcite and aragonite in the Baltic Sea. The average seasonal amplitude of Ω is unusually high in the Baltic Sea (range of about 4) in comparison to open ocean locations (range of between 1 and 2), and varies from about 1 to 5 for calcite and from 0.5 to 2.5 for aragonite with minimum in winter and maximum in summer (Findlay et al. 2007). Such low winter values do not occur in oceanic surface waters at similar latitudes, which are significantly oversaturated (Ω > 1) with regard to both calcite and aragonite throughout the year (Tyrrell et al. 2007).

### Species

Populations of the common mussel *Mytilus* sp. are key elements of intertidal and subtidal hard-bottom communities from the temperate to subarctic coastal zones of the Northern Hemisphere (Väinölä and Strelkov 2011). For the purpose of this investigation, we focus on *M. trossulus* Gould (Mollusca, Bivalvia). This mollusc occurs on various types of substrates including rocky surfaces, wrecks, pier pilings and harbour walls (Rainbow et al. 1999); hence, it is easy to collect. *M. trossulus* can be the dominant member of a diverse community of invertebrates such as barnacles, nematodes, polychaetes, bryozoans, gastropods or hydrozoans. It is a filter feeder preying mainly on phytoplankton (Rainbow et al. 2004; Lauringson et al. 2013). At the same time it is an important component in the diet of many predators in the Gulf of Gdansk, especially for flatfish, cod and crab *Eriocheir sinensis* (Wojcik et al. 2015). It is a temperate and coldwater species, becoming more abundant in the northern reaches of its range (Ricketts and Calvin 1971), where it encounters optimum growth temperature of 10–20 °C (Haderlie and Abbott 1980). The physiology of *M. trossulus* in the Gulf of Gdansk is no doubt under the control of low salinity and seasonal variability. Generally, *M. trossulus* grows up to 10 cm in height (Gofas 2004), yet in the estuarine environment of the Gulf of Gdansk, it reaches a maximum length of up to about 50 mm (Abramowicz personal comm.). *M. trossulus* is better adapted to variable, particularly lower, salinity than other *Mytilus* species. Riisgård et al. (2013) revealed that the growth rates of *M. edulis* and *M. trossulus* under the same conditions with salinity around 20 are nearly identical. Both species have the ability to grow fast under optimal conditions and to reduce growth rate due to decreasing salinity, but only *M. trossulus* is able to completely acclimatise its filtration rate to salinities below 7 over extended time periods (Riisgård et al. 2013, 2014). The annual thermal amplitude in the gulf makes the environmental conditions leading to mussels highest biomass production increase from spring which reaches a maximum in June, then decreases through autumn and winter. Such patterns could be linked to reduction or lack of food availability in colder seasons or to gonad development after spawning. Overlapping changes in metabolic rate result in winter hold up of absorbed food at the expense of reduction in vital processes (Pierścieniak et al. 2010).

The shell of *M. trossulus* is bimineralic and consists of two calcium carbonate layers: outer calcite and inner aragonite in variable proportions between individuals (Eisma et al. 1975; Dalbeck 2008; Zhang and Zhang 2006).

### Sample collection and preparation

Bivalves were collected from 36 m depth in the Gulf of Gdansk in May 2013 at GN station (Fig. 1), which is the site used by researchers over many years to obtain samples of *M. trossulus* (Szefer and Szefer 1990; Rainbow et al. 2000; Rainbow et al. 2004). Each of the individuals was from a single population, thereby was subjected to the same environmental conditions during development, reducing the number of variables that may influence the mineralogy and chemical composition of a shell. The water temperature at the bottom during sample collection was 3.1 °C and the salinity was 7.3. Sample was obtained using a Van Veen grab sampler from RV *Oceanograf-2*.

**Fig. 2:**
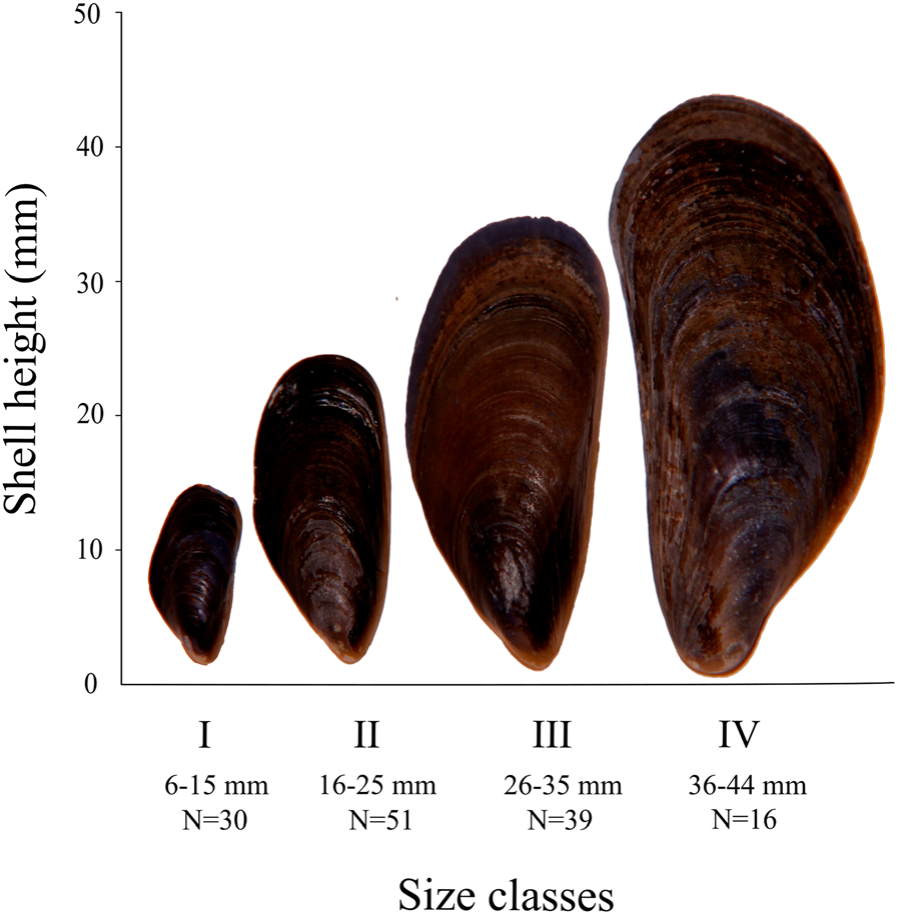
The ranges of each size classes of *M. trossulus* shells from the Gulf of Gdansk. *N* is a number of analysed individuals

All mussels were collected alive and transported to the laboratory. One hundred thirty six individuals were selected for further analysis. Mussels from the natural environment are abundantly covered with overgrowing flora and fauna, mainly barnacles and bryozoans, which would disrupt the results. Therefore, after the removal of soft tissues, every individual was viewed under a stereoscopic microscope to check for the unwanted organisms which, if present, were removed. Each shell was cleaned by sonication for 30 min in deionized water and air-dried. The periostracum was removed using the scalpel. By measuring along the shell height, using the calliper with an accuracy of ±1 mm, individuals were divided into four size classes: I (6–15 mm), II (16–25 mm), III (26–35 mm) and IV (36–44 mm) (Fig. 2), reflecting the range in size from juvenile to adult for the southern Baltic Sea population.

From all individuals, one valve was ground into powder using an agate pestle and mortar and subjected to mineralogical analysis to evaluate the calcite and aragonite content. To estimate the chemical composition of *M. trossulus* shells, the second valve from six individuals at each size class was selected for elemental analysis, weighed and grounded into powder using an agate pestle and mortar. The aliquots (5–320 mg) of powdered samples were weighed using a 5-digit analytical balance into a 15-ml plastic tube (Sarstedt), dissolved in 1.5 ml concentrated nitric acid (HNO_3_, Sigma-Aldrich, Trace SELECT^®^ for trace analysis), 1.5 ml ultra-pure water (18.2 mohm cm^−1^) and 0.3 ml 30% hydrogen peroxide (H_2_O_2_, Merck Suprapur^®^ grade) for 24 h at 70 °C and made up to 15 ml by weight with ultra-pure water. After mixing the solution and allowing enough time for the undigested organic material to settle, the top 10 ml of the sample solution was collected for the ICP-AES and ICP-MS analysis.

### Mineralogical and elemental analyses

Mineralogical analyses were conducted using a high-precision Enraf-Nonius X-ray diffractometer with a position-sensitive detector and a cobalt X-ray source (XRD-PSD) at the Natural History Museum, London. Powdered samples were placed on a circular glass, forming a flat, homogeneous surface and loaded into a deep-well circular rotary mount. This reduced the error resulting from preferential orientation of crystallites, being the cause of randomly generated XRD patterns. Tube operation conditions were 40 kV and 40 mA. A primary germanium 111 monochromator in combination with slit settings of 0.14 × 5 mm was used to limit the X-ray beam to pure Co Ka_1_ radiation. Diffracted intensities were collected simultaneously over a 2-Theta range of 120°. Time adopted for each sample analysis was 15 min. The amount of each polymorph in *M. trossulus* shells was determined by fitting peak intensities compared to standard patterns generated from 100% aragonite (BM 53533) and 100% calcite (ground Iceland spar). The error associated with this method was obtained by testing sample with a known calcite to aragonite ratio and it was estimated to be within ±1%. Patterns were collected and performed using Difgrab™ software. The software has capabilities of not only mineral determination but also peak shifts and intensity analysis. Difgrab™ has been specially developed in-house for data collection for Enraf-Nonius XRD at Natural History Museum in London. The mineralogical results were expressed in percentage, assuming that the content of calcite + the content of aragonite = 100%.

Major and trace element composition of *M. trossulus* shells were determined at the Natural History Museum, London, using a Thermo iCap 6500 Duo inductively coupled plasma - atomic emission spectrometer (ICP-AES) for Ca, Mg, Na, Sr and Mn, and an Agilent 7700x inductively coupled plasma mass spectrometer (ICP-MS) for Ba, Cd, Co, Cu, Fe, Ni, Pb, U, V, Y and Zn. Calibration of ICP-AES analysis was performed using solutions that were matrix-matched to the high calcium concentration in the samples. Multiple wavelengths for each element were recorded and line selection was made accounting for the suitability of the wavelength to the sample concentrations. The accuracy and reproducibility of the analyses were checked using two calcium carbonate-rich certified reference materials (CRMs): JLs-1 Limestone and JDo-1 Dolomite (both from the Geological Survey of Japan) prepared by total digestion method (using hydrofluoric acid). The reference materials were diluted to match the concentrations of Ca in sample solutions. Ca, Mg and Sr concentrations were found to be within the uncertainty (1 standard deviation) of the reported values (Imai et al. 1996).

Limits of quantification (LOQ) in solution for ICP-MS were generally determined as a concentration corresponding to ten times standard deviation of the signal obtained by analysing 5% HNO_3_ solution (6–7 times) in each individual run. If a preparation blank for a particular element (e.g. Zn) was higher than LOQ, the operational LOQ in the sample was determined either five times the concentration in the preparation blank or ten times standard deviation of the preparation blank (where at least three individual blanks were available in a single digestion batch), whichever is greater. ICP-MS was run in helium (He) mode (5 ml min^−1^ He, 99.9995% purity) for most trace elements (V, Mn, Fe, Co, Ni, Cu and Cd) to minimize the molecular interferences from plasma and solution components and Ca from samples; however, ^59^Co^+^ and ^60^Ni^+^ signals still suffered from potential interferences from ^43^Ca^16^O^+^ and ^44^Ca^16^O^+^, respectively. Pure Ca solutions at the same concentrations of Ca as the samples were run periodically to check on the level of potential interferences and based on the results the operational LOQ was set up for Co at 0.06 mg kg^−1^and for Ni at 0.58 mg kg^−1^ in the samples containing 35% of Ca. The accuracy and reproducibility was checked by periodic analyses of JLs-1 and JDo-1. The results obtained for all elements (Table 1) were within the uncertainty (2.5 SD) of the recommended values (Imai et al. 1996). Accuracy of Pb determination cannot be checked using these CRMs because of the large spread of reference values probably due to insufficient homogeneity of Pb distribution in these samples. Based on the analyses of CRMs and matrix-matched solutions, the maximum analytical error for the typical range of concentrations in the shells can be estimated (in relative percentage) as 1.5% for Ca, Mg and Sr; 3% for Fe and Ba; 20% for Co, Ni, Cu, Zn and U; and 4–10% for all other elements. The results were expressed in weight percent and milligrams per kilogram (ppm) of the shell dry weight (Tables 2, 3 and 4). Mg and Sr contents were converted to molar concentrations, normalized to Ca concentration and expressed as element/Ca (mmol/mol) (Table 4).

**Table 1:** Bulk concentrations of major, minor (in wt% as oxides) and trace elements (in mg kg^−1^ as elements) obtained in this study for the CRMs JLs-1 (Limestone) and JDo-1 (Dolomite)

Element (mg kg^−1^)	JLs-1				JDo-1			
	This work (*N* = 4)		Imai et al. 1996		This work (*N* = 4)		Imai et al. 1996	
	Average	SD	Average	SD	Average	SD	Average	SD
CaO (wt%)	55.4	0.4	55.1	0.3	33.7	0.2	33.9	0.4
MgO (wt%)	0.60	0.01	0.60	0.06	18.7	0.15	18.5	0.35
Sr	288	5	295	15	115	2	116	6
V	3.6	0.3	3.6	0.1	3.8	0.3	3.1	0.9
Mn	17.5	0.8	16.2	4.2	52.3	2.7	50.9	4.2
Fe	113	2.6	117	45	144	4.6	145	28.6
Co	0.066	0.006	0.083	0.044	0.150	0.007	0.168	0.033
Ni	<0.58	–	0.36	0.07	3.09	0.15	2.9	0.72
Cu	0.39	0.02	0.27	0.08	1.82	0.21	1.41	0.18
Zn	<8	–	3.2	0.7	39.1	1.9	35.4	1.6
Y	0.25	0.02	0.22	0.047	11.1	0.6	10.3	0.7
Cd	0.17	0.006	0.16	0.005	0.63	0.02	0.64	0.13
Ba	478	13	476	45	6.1	0.05	6.1	0.53
Pb	0.44	0.24	0.22–1.21^a^	–	0.48	0.09	0.19–1^a^	–
U	1.84	0.25	1.75	0.29	0.90	0.14	0.86	0.14

*SD* standard deviation, *N* number of replicate analyses

^a^The range of reference Pb values is given to show the potential heterogeneity of samples with respect to Pb

**Table 2:** Concentrations of major, minor and trace elements obtained in this study using ICP-AES and ICP-MS methods

Element	Ca	Fe	Mg	Na	Sr	Ba	Cd	Co	Cu	Mn	Ni	Pb	U	V	Y	Zn
Unit	wt%	wt%	wt%	wt%	wt%	mg kg^−1^	mg kg^−1^	mg kg^−1^	mg kg^−1^	mg kg^−1^	mg kg^−1^	mg kg^−1^	mg kg^−1^	mg kg^−1^	mg kg^−1^	mg kg^−1^
Method	ICP-AES	ICP-AES	ICP-AES	ICP-AES	ICP-AES	ICP-AES	ICP-MS	ICP-MS	ICP-MS	ICP-AES	ICP-MS	ICP-MS	ICP-MS	ICP-MS	ICP-MS	ICP-MS
Size class																
I	31.8	0.004	0.107	0.197	0.111	<11.1	0.160	0.084	34.8	38.9	1.94	3.88	0.111	1.890	0.096	42.8
I	33.8	0.002	0.121	0.212	0.104	<8.6	0.101	0.072	26.6	31.8	1.89	3.42	0.077	1.400	0.087	27.3
I	33.2	<0.005	0.116	0.291	0.120	<24.5	0.165	0.188	58.3	<73.6	4.78	6.26	0.115	2.300	0.150	42.4
II	35.4	0.003	0.140	0.198	0.120	9.0	0.689	0.560	25.7	62.0	1.66	3.08	0.069	1.990	0.066	42.5
II	35.5	0.003	0.116	0.220	0.130	10.7	0.053	0.078	20.4	58.7	1.01	1.35	0.049	0.694	0.074	27.1
II	35.4	0.004	0.117	0.229	0.127	18.8	0.157	1.085	24.4	51.9	1.16	1.31	0.129	0.520	0.073	–^a^
II	35.6	0.009	0.109	0.207	0.120	16.5	0.190	<0.06	16.2	48.6	1.12	1.04	0.120	0.941	0.077	53.8
II	35.9	0.004	0.102	0.219	0.122	32.4	0.150	0.071	21.3	52.2	1.00	1.05	0.116	0.879	0.088	54.8
II	38.1	0.010	0.100	0.227	0.117	14.8	0.083	0.185	10.2	198^b^	0.59	<0.83	0.030	0.628	0.085	29.7
III	38.7	0.002	0.122	0.210	0.110	10.5	0.028	<0.06	6.3	33.4	<0.58	<0.65	0.022	0.206	0.040	12.2
III	38.5	0.046	0.128	0.176	0.114	19.4	n/a	n/a	n/a	189^b^	n/a	n/a	n/a	n/a	n/a	n/a
III	31.0	0.021	0.101	0.167	0.112	10.2	0.028	<0.06	7.6	37.2	<0.58	<0.46	0.029	0.429	0.080	13.6
III	34.5	0.001	0.098	0.201	0.110	10.3	0.103	0.065	13.4	63.6	0.73	0.75	0.066	1.070	0.055	34.3
III	38.1	0.102	0.137	0.186	0.130	25.8	0.082	0.157	8.1	77.8	<0.58	<0.63	0.033	0.532	0.168	20.9
III	35.2	0.027	0.108	0.167	0.116	13.9	0.039	0.078	10.2	65.3	<0.58	<0.51	0.024	0.368	0.086	15.1
IV	37.7	0.006	0.104	0.196	0.109	9.6	0.031	0.085	7.3	54.3	<0.58	<0.78	0.033	0.601	2.755	12.6
IV	34.0	0.059	0.100	0.227	0.137	16.9	0.030	0.087	8.0	39.3	0.78	1.07	0.021	0.411	0.112	27.4
IV	30.4	0.050	0.125	0.197	0.128	28.3	0.105	0.072	12.1	49.3	1.22	1.54	0.051	0.386	0.886	26.5
IV	25.2	0.012	0.061	0.147	0.103	15.6	0.023	0.076	3.4	48.1	<0.58	<0.3	0.025	0.290	0.086	11.0
IV	28.1	0.015	0.067	0.170	0.114	16.6	0.032	0.087	4.9	46.0	<0.58	0.33	0.026	0.291	0.101	15.6
IV	38.1	0.016	0.105	0.180	0.125	13.6	0.034	0.064	10.9	46.8	<0.58	<0.61	0.018	0.344	0.067	19.8

The results with less-than sign (<) were not used in the calculations

*n*/*a* not analysed

^a^Sample contaminated with Zn in the laboratory and the result was not used in the calculations

^b^Outliers

**Table 3:** Average concentrations of major, minor and trace elements in *M. trussulus* shells

Element	*N*	Average	Minimum	Maximum	SD
wt%					
Ca	21	34.5	25.2	38.7	3.6
Na	21	0.201	0.147	0.291	0.031
Sr	21	0.118	0.103	0.137	0.009
Mg	21	0.109	0.061	0.140	0.019
Fe	20	0.020	0.001	0.102	0.026
mg kg^−1^					
Mn	18	50.3	31.8	77.8	12.1
Zn	19	27.9	11.0	54.8	13.9
Cu	20	16.5	3.4	58.3	13.0
Ba	18	16.3	9.0	32.4	6.7
V	20	0.809	0.206	2.300	0.619
Y	20	0.262	0.040	2.755	0.614
Co	17	0.182	0.064	1.085	0.261
Cd	20	0.114	0.023	0.689	0.146
U	20	0.058	0.018	0.129	0.039

Only elements with more than 85% of concentration values above LOQ are shown

*N* number of analysed individuals, *SD* standard deviation of the average

**Table 4:** Concentrations of elements, for which no statistically significant differences were found between the size classes of *M. trossulus*

Element	Size class	*N*	Average	Minimum	Maximum	SD	*p* value
Ca (wt%)	I	3	32.9	31.8	33.8	1.1	0.14
	II	6	36.0	35.4	38.1	1.1	
	III	6	36.0	31.0	38.7	3.0	
	IV	6	32.2	25.2	38.1	5.2	
Sr (wt%)	I	3	0.112	0.104	0.120	0.008	0.25
	II	6	0.123	0.117	0.130	0.005	
	III	6	0.115	0.110	0.130	0.008	
	IV	6	0.119	0.103	0.137	0.013	
Mg (wt%)	I	3	0.115	0.107	0.121	0.007	0.33
	II	6	0.114	0.100	0.140	0.015	
	III	6	0.116	0.098	0.137	0.016	
	IV	6	0.094	0.061	0.125	0.025	
Y (mg kg^−1^)	I	3	0.111	0.087	0.150	0.034	0.10
	II	6	0.077	0.066	0.088	0.008	
	III	5	0.086	0.040	0.168	0.050	
	IV	6	0.668	0.067	2.755	1.071	

*p* value is the significance level of the Kruskal–Wallis tests

*N* number of analysed individuals, *SD* standard deviation of the average

### Statistical analysis

During this study, two main hypotheses were tested. The null hypothesis (H_0_) assumed that there are no statistically significant differences between the elemental and mineralogical composition of *M. trossulus* shells in four size classes. The alternative hypothesis (H_1_) assumed statistically significant differences. In order to verify the assumptions, the concentration of 16 elements, Ca, Na, Mg, Sr, Fe, Mn, Zn, Cu, Ba, V, Ni, Pb, Y, Cd, Co and U, and the proportion of calcite to aragonite in shells were used. Statistics for size classes were made only for the elements with all the values above the LOQ (limit of quantification), that is for Ca, Mg, Na, Sr, Cd, Cu, U, V, Y and Zn. The data did not improve normality (Shapiro–Wilk test); therefore, variability of elements and the ratio of calcite to aragonite between size classes were identified using one-way nonparametric Kruskal–Wallis tests for multiple independent groups. In the case of Pb, two size classes were tested by Mann–Whitney *U* test for two independent groups. The significance level of the tests (*p* value) was set at 0.05. If the *p* value was smaller than the significance level, the null hypothesis was rejected and the alternative hypothesis was accepted as true. This allowed us to test whether the size of the shells affects the composition of the shells. All statistical procedures were run using STATISTICA ver. 10 (StatSoft Inc.).

## Results and discussion

### Mineralogical and elemental description of *M. trossulus* shells

**Fig. 3:**
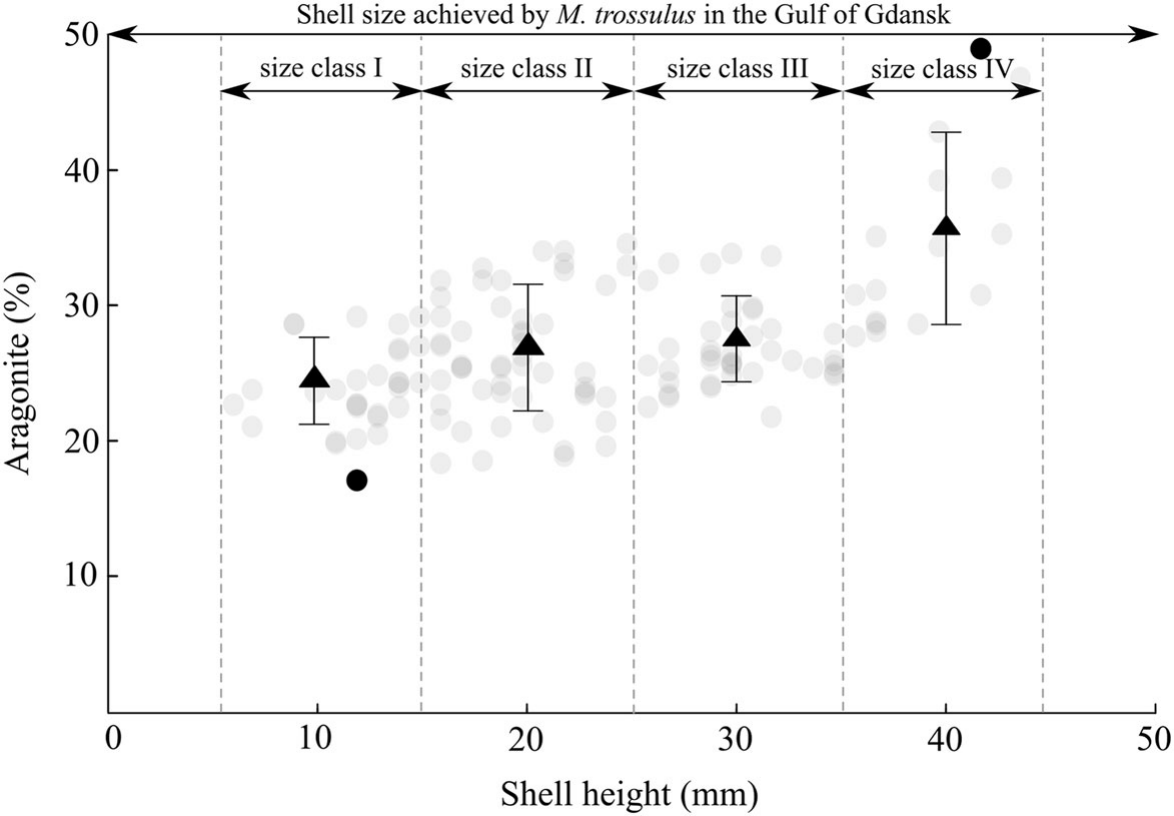
The variability of aragonite content depending on the height of the *M. trossulus* shells from Gulf of Gdansk in the Baltic Sea. Data points show all analysed individuals, black points correspond to minimum and maximum values. *Triangles* show average values; *error bars* indicate standard deviation

Analysed shells of *M. trossulus* are built of two polymorphs of calcium carbonate: calcite and aragonite, with calcite being a dominant form (Fig. 3). Recent studies found CaCO_3_ polymorph selection might be temperature-dependent, with aragonite precipitation at high temperature and calcite precipitation at low temperature (Cohen and Branch 1992; Kuklinski and Taylor 2009; Ramajo et al. 2015; Krzeminska et al. 2016). Lowenstam (1954) showed that Mytilidae species occupying subtropical and tropical niches may precipitate 100% aragonite shells, bimineralic *Mytilus* sp. species living in mean environmental temperatures above 22 °C lay down shell with dominant aragonite content, whereas at lower temperatures, the shell composition is mainly dominated by calcite. This was concluded based on the correlation of the maximum aragonite content with the mean yearly temperature in different locations, from Greenland (*M. edulis*, 45%, 3.2 °C) to Philippines (*M. smaragdinus*, 100%, 28.5 °C). In this study, in a temperate climate, the highest content of aragonite for *M. trossulus* is 49.6% (Fig. 3) with mean yearly water temperature about 8 °C, supporting the temperature dependence. However, this is probably not the only factor shaping calcium carbonate polymorphism. Parameters such as salinity, pH or elemental composition of the environment indirectly determine the mineralogy of shells (Dissard et al. 2010). Dodd (1963) revealed an inverse relationship between salinity and shell aragonite content, what was confirmed by Zhong and Mucci (1989); Malone and Dodd (1967) or Salisbury et al. (2008). However, Ramajo et al. (2015) observed that aragonite levels in shells of marine snail *Concholepas concholepas* along the Chilean coast with opposite temperature and salinity gradients are under temperature control, establishing that temperature is the major factor controlling aragonite to calcite ratio. In the modern world, including the Gulf of Gdansk (Fig. 1), changes in atmospheric pCO_2_ also potentially contribute to shifts in skeletal mineralogy (Zhuravlev and Wood 2009; Lee and Morse 2010). With rising pCO_2_ and following reductions in carbonate saturation state, the precipitation of calcite, a mineral with higher stability and lower solubility, seems to be favoured (Morse et al. 2006; Zhuravlev and Wood 2009) due to its higher resistance to corrosion. In the bimineralic system of *M. trossulus* shells from the Gulf of Gdansk, it is likely that outer calcitic layer serves as a protective shield for the inner aragonite layer, and the variability in their proportion on a global scale seems to be the ability of mussels to adjust to the environment. Ocean acidification studies have also reported that species exposed to short periods of reduced pH present different mechanisms of acid–base regulation (Hendriks et al. 2015), usually linked with a metabolic depression (Dupont et al. 2010; Stumpp et al. 2011) and reallocation of energy for essential biological processes such as reproduction and growth (Gattuso and Hansson 2011). As calcite is less energetically costly than aragonite for the organism to precipitate (Ramajo et al. 2015), any fluctuations in metabolic demand including those related to growth and reproduction might stimulate dominance of a given mineral in the shell structure. Palmer (1992) found that for a shell with only 1.5% organic matter, this organic material costs 22% of the total energy of the shell production. In a shell with 5% organic matter, this cost rises to 50%. Aragonite requires more organic material in construction; thus, it is more expensive to precipitate. Therefore, secretion of this polymorph might be regulated by metabolic demand of the organism. As the Gulf of Gdansk is a very seasonal system, the food availability with abundance peaks in spring and summer which could be also a potential factor responsible for observed mineral proportions dominated by calcite (Fig. 3). A reduced food base in colder periods might force *M. trossulus* to minimize all the vital processes (Pierścieniak et al. 2010) and to sustain on accumulated food energy. While warmer seasons favour aragonite layering, annually, domination of calcite appears to be an adaptation to the ongoing environmental conditions. The aragonite represents a carbonate polymorph that is mechanically stronger, denser and more elastic than calcite (Taylor and Layman 1972; Zuschin et al. 2003). As such, aragonite, due to its mechanical design, is considerably more resistant to crushing and boring by predators when compared with calcite and is also more resistant to hydrodynamic erosion including storms or waves (Jackson et al. 1988; Gray and Smith 2004). Yet, the site, where our specimens were collected, is rather deep (36 m), hence protected from wave action and, generally, from stormy events. Also, *Mytilus* species in the Gulf of Gdansk do not have many competitors for space or food as it is one of a few bivalves occurring on firm substrate in the area. Thus, all the adaptations which may lead to a change in mineral proportion as a result of hydrodynamics or protection against competition or predation can be considered insignificant.

Different ions, replacing Ca^2+^ in the calcium carbonate structure, are incorporated into the shell during its formation. The availability and bioaccumulation rate of ions from sea water seem to be a function of environmental and biological factors (Rainbow 1990; Wright 1995; Wang and Fisher 1997). Thus, different habitats, species or even individual specimens at a different stages of development, may present different patterns of metal uptake. This is the reason why each potential bioindicator should be examined from several angles. Generally, it is established that the activity of free ions rather than total metal concentration determines the biological availability of dissolved metals. Both low salinity and low pH increase free ion activity of several metals (Fritioff et al. 2005), which is especially important in brackish habitats. At salinities below 10, the decrease of Ca^2+^ concentration in water promotes an influx of ions such as Cd^2+^, Co^2+^, Cu^2+^ or Zn^2+^ by reducing competition for the same sites in the crystal lattice (Wright and Zamuda 1987). This tendency is enhanced by the fact that the concentration and bioavailability of Sr^2+^ and Mg^2+^, major substituents of Ca^2+^ in biocarbonates, is usually much lower in fresh water than in seawater (Dettman et al. 2004; Poulain et al. 2015). Furthermore, lower salinity means lower level of chloride ions (Cl^−^), forming complexes with metals; hence, their availability is higher in brackish than in marine environments (Fritioff et al. 2005). In the shells of *M. trossulus* from the southern Baltic Sea, 16 elements were measured: Ca, Fe, Mg, Na, Sr, Ba, Mn, Cd, Co, Cu, Ni, Pb, U, V, Y and Zn (Table 2) with mean concentrations ranging from 34.5 ± 3.6 wt% for Ca to 0.058 ± 0.039 mg kg^−1^ for U (Table 3). Due to large differences in the concentrations, the results for Ca, Na, Sr, Mg and Fe were expressed as weight percent, while for Mn, Zn, Cu, Ba, V, Y, Co, Cd and U as milligrams per kilogram of the shell dry weight (Table 3). The Baltic Sea region has been sporadically examined for elemental levels in shells of *Mytilus* species. Szefer et al. (2002) and Protasowicki et al. (2008) studied shells of *Mytilus* sp. from the Gulf of Gdansk for identification of coastal areas exposed to metallic contaminants. In both cases, levels of metals were lower for Zn, Cu and Fe, higher for Mn and similar for V, compared with this study (Table 3). The differences may be related to variations in environmental metal concentrations, which could be the result of intermittent terrestrial run off or sediment resuspension, caused by hurricanes or storms. Although those events dominate in the Gulf of Gdansk during winter and early spring, when the growth and precipitation of mussels is limited, yet they can alter the biogeochemistry of the water column for several months (Paerl et al. 2001; Gillikin et al. 2005b).

### Size-dependent variations in mineralogy and elemental constitution of *M. trossulus* shells

The size of the shells had a statistically significant impact on the content of the produced polymorphs. The content of aragonite increased with increasing body size (Kruskal–Wallis test: H_3,136_ = 35.98, *p* < 0.0001), ranging from 23.8 ± 3.2% in the size class I to 35.0 ± 7.1% in the size class IV (Fig. 3). Generally within genus *Mytilus*, as suggested by Dodd (1966), the percentage of aragonite in the bimineralic shells seems to be correlated with seasonal temperature variability. Dodd (1963) also noted that the mineralogy of small (less than 15 mm long) shells of *Mytilus californianus* is not temperature-dependent while larger specimens show a positive correlation between the percentage of aragonite and temperature. Similarly, variations in aragonite content within shells of *Mytilus* sp. were shown to change with geographical location, decreasing towards higher latitudes (Dodd 1963). In our study, *M. trossulus* shells representing all size classes were collected in May, when the water temperature was 3.1 °C. However, the Gulf of Gdansk is under the influence of seasonal variability (Uścinowicz 2011). Considering that *M. trossulus* reproduce from late spring to early autumn, and in the study area mainly in August (Dziubińska and Janas 2007), individuals from size class I briefly lived in the warm summer season in comparison with other size classes. This may explain the smallest content of aragonite in the youngest shells (Fig. 3). Seasonality determines not only temperature but also, inter alia, food availability, salinity, light and pH, and all these parameters control the growth rate and calcification of bivalves (Wong and Levinton 2004; Berge et al. 2006; Hiebenthal et al. 2012), so that the summer growth maximum results in aragonite accumulation. In the context of ontogeny, the key variable during the development of living organisms is metabolic rate (Hahn et al. 2014). As mentioned above, calcite precipitation is less energetically costly (Ramajo et al. 2015); therefore, younger individuals with a higher metabolic rate, have shells built mainly with calcite (Fig. 3), placing energy in the physiological processes. Ramajo et al. (2015) suggested that juvenile individuals of snails *C. concholepas* may favour the precipitation of calcite, as less soluble and thermodynamically more stable form than aragonite, to make their small thin shells more resistant against corrosion and dissolution. Furthermore, it is possible that, if environmental factors promote erosion of the outer calcitic layer, older shells are left with a high aragonite content.

**Fig. 4:**
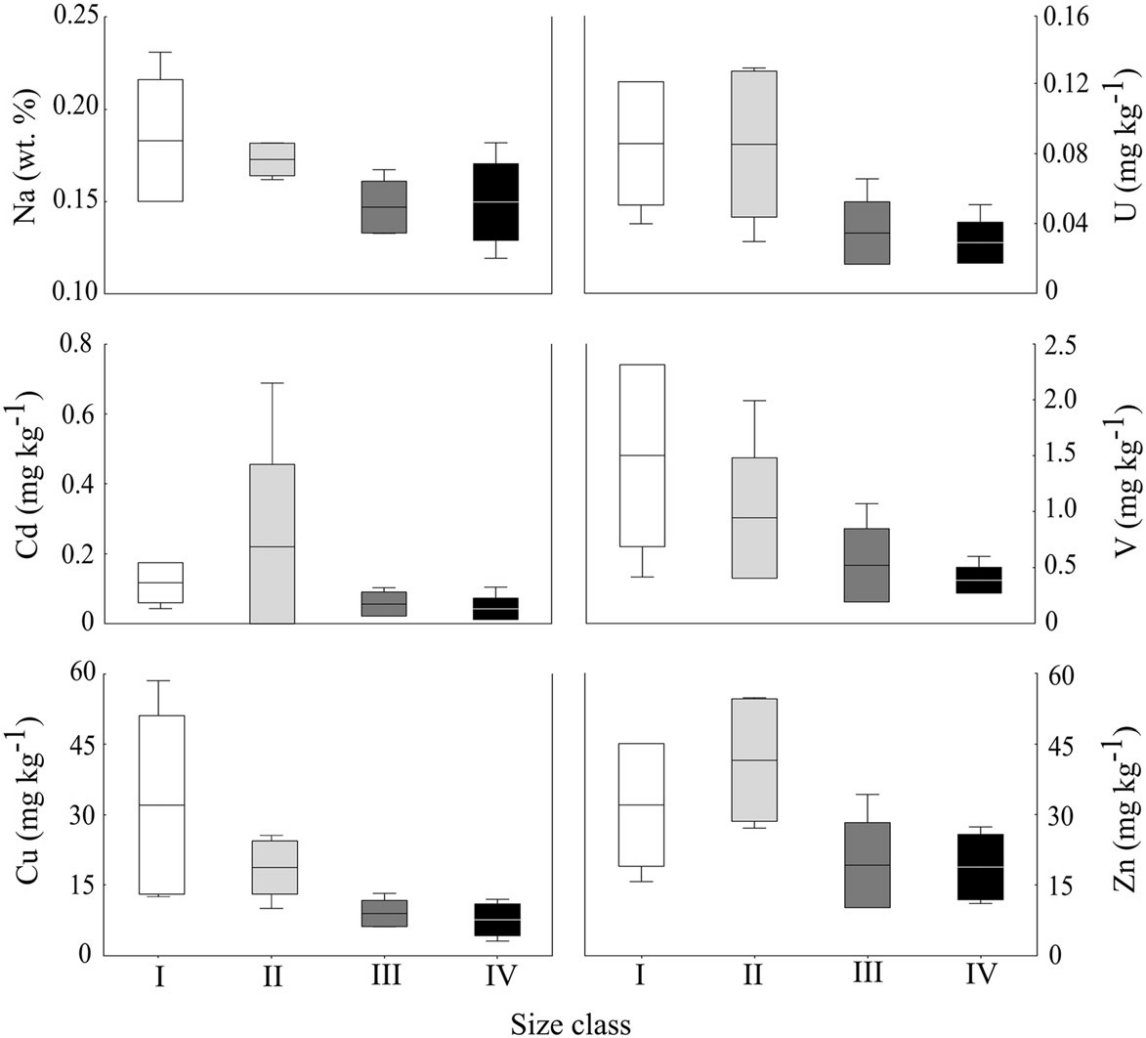
Box plots showing concentrations of elements (wt% and mg kg^−1^) with statistically significant differences between the size classes in *M. trossulus* shells (Kruskal–Wallis test). Boxes show the standard deviation around mean (*middle lines*). *Bars* indicate ranges of concentration values

This study furthermore confirms that the given size of the individual is associated with a certain range of concentrations of elements in shells (Fig. 4). The nonparametric Kruskal–Wallis tests used in this study revealed a significant correlation (*p* < 0.05) between the element concentrations and shell height ranges for six out of ten selected elements: Na (H_3;21_ = 8.99, *p* = 0.03), Cd (H_3;20_ = 10.54, *p* = 0.01), Cu (H_3;20_ = 13.86, *p* = 0.003), U (H_3;20_ = 11.14, *p* = 0.01), V (H_3;20_ = 12.16, *p* = 0.01) and Zn (H_3;19_ = 10.42, *p* = 0.02). In all these cases, the element concentrations decrease with increasing shell size (Fig. 4). Eight out of 20 results for Pb were below the LOQ, but its concentrations appreciably decrease with shell growth (Table 2), which was confirmed by Mann–Whitney *U* test (*p* = 0.037). High variability among different individuals of the same species confirms the general assumption that bioaccumulation changes with organism size as well. The dependence of metal influx rates on size is commonly explained by size dependence of the metabolic rate (Newman and Heagler 1991; Wang and Fisher 1997), which, generally, is faster in the smaller individuals than in the larger ones. Therefore, the intensity of the metal uptake could be greater in smaller bivalves than in larger ones for the same species and habitat (Lee et al. 1998). The bimineralic nature of *M. trossulus* and the increasing content of aragonite throughout the size classes (Fig. 3) is probably one of the factors contributing to the elemental variation. Differences in the trace element concentrations between the two polymorphs of CaCO_3_ are in agreement with the results obtained by other authors (England 2005; Dalbeck 2008; Iglikowska et al. 2017). Calcite and aragonite have the same chemical composition, but different crystal structures. Hence, trace elements with ionic radii less than Ca^2+^ (e.g. Mg^2+^, Cu^2+^, Zn^2+^, U^4+^, Cd^2+^, V^3+^, Y^3+^) generally accumulate preferably in calcite, while metal ions larger than Ca^2+^ (e.g. Sr^2+^, Pb^2+^, Na^+^) tend to become preferentially incorporated in aragonite (Langston et al. 1998). In our study, the concentration of Na, Cu, Zn, U, Cd and V decrease with increasing shell size (Fig. 4). The ontogenetic development of *M. trossulus* is accompanied by decreasing content of calcite in favour of aragonite (Fig. 3), which may be linked with the reduced accumulation of Cu, Zn, U, Cd and V. However, the concentration of Na decreases despite the growing aragonite crystal lattice, and the concentration of Mg and Sr do not seem to depend on the shell length (Tables 4 and 5). Both Mg and Sr are important elements in context of geochemical, paleooceanographic and environmental monitoring studies based on bivalves. Many authors suggested their potential usage as temperature proxy (Dodd 1965; Klein et al. 1996b). However, the majority of previous studies have concluded that the Sr/Ca and Mg/Ca ratios of bivalves depend on the growth rates or other physiological parameters instead of water temperatures. Some authors have assumed an indirect correlation between water temperatures and shell Sr/Ca and Mg/Ca values because the metabolism is influenced by the ambient environment (Gillikin et al. 2005a; Freitas et al. 2006; Elliot et al. 2009, Poulain et al. 2015). Yet, there is no certainty whether environmental variables can be reconstructed from metal proxy excluding vital effect processes (Schöne et al. 2011). Common heavy metal pollutants Cd, Cu, Zn, V and U are often used as indicators of human impact on marine environment (Lindström 2001; Reiss et al. 2004). The concentration of each element decreases throughout the size classes of *M. trossulus* (Fig. 4). Potential reasons for this may include changes in anthropogenic input of metals over the growth period, seasonal variability in water parameters of the Baltic Sea or physiological changes of mussels during development. The intensity of heavy metals discharge into the Gulf of Gdansk depends on a number of events such as the flow rate of rivers associated with rainfall or melting, storms and water mixing or current scale of pollution. Subsequently, the availability of free ions is controlled, inter alia, by temperature or salinity (Fritioff et al. 2005), and these change between seasons and even years. That is why the environmental bioavailability of elements may vary throughout the life of *M. trosullus*.

**Table 5:** Mg/Ca and Sr/Ca ratio (mmol/mol) in the shells of *M. trussulus* in four size classes

Element	Size class	*N*	Average	SD	Minimum	Maximum
Mg/Ca	I	3	5.34	0.83	4.12	5.90
	II	6	5.23	0.75	4.34	6.51
	III	6	5.29	0.41	4.70	5.92
	IV	6	4.77	1.05	3.91	6.78
Sr/Ca	I	3	1.53	0.12	1.41	1.65
	II	6	1.56	0.10	1.40	1.68
	III	6	1.47	0.13	1.30	1.66
	IV	6	1.72	0.25	1.32	1.92

*N* number of analysed individuals, *SD* standard deviation of the average

## Conclusions

All examples considered in this study, although to some extent speculative, suggest that elemental concentrations in the shells are coupled with the mineralogical composition; thus, the mechanisms controlling their concentration, at least for some of the elements, might be similar. The crystal structure of the polymorph produced can play an important role in determining the uptake of certain elements, and disorders in the crystal lattice can facilitate incorporation of some other ions. However, this relationship seems to be very complex, and studies in other regions and of other species (Dodd 1967; Cronin et al. 2005; Gillikin et al. 2005a; Strasser et al. 2008; Izumida et al. 2011) suggest that it can be controlled by the external environmental parameters or by certain physiological mechanisms specific for the organism, which can lead to observed ontogenetic effects. Consequently, this study indicates that, when using mineralogical and chemical composition of the shells as environmental indicators, the size of the organisms should be taken into account. In order to minimize the bias in mineralogical or chemical composition related to the size of the shell, individuals of the same or very similar dimensions should be selected for the analysis.

Lack of statistically significant changes in the concentration of Ca, Sr, Mg and Y throughout size classes of *M. trossulus* determined in this study may suggest that these elements can be used as environmental proxies regardless of the shell size. Yet, in the reconstruction of the environmental parameters, comparison of chemical compositions of whole shells of the same size will most likely introduce the least bias into the interpretation.

## References

[CR1] Addadi L, Weiner S (1992). Control and design principles in biological mineralization. Angewandte Chemie International Edition.

[CR2] Andral B, Stanisiere JY, Sauzade D, Damier E, Thebault H, Galgani F, Boissery P (2004). Monitoring chemical contamination levels in the Mediterranean based on the use of mussel caging. Marine Pollution Bulletin.

[CR3] Arai T, Maeda M, Yamakawa H, Kamatani A, Ohji M, Miyazaki N (2003). Uptake and elimination of trace metals in shells of abalones *Haliotis* spp. Bulletin of Environmental Contamination and Toxicology.

[CR4] Belcher AM, Wu XH, Christensen RJ, Hansma PK, Stucky GD, Morse DE (1996). Control of crystal phase switching and orientation by soluble mollusc shell proteins. Nature.

[CR5] Berge JA, Bjerkeng B, Pettersen O, Schaanning MT, Øxnevad S (2006). Effects of increased sea water concentrations of CO_2_ on growth of the bivalve *Mytilus edulis* L. Chemosphere.

[CR6] Bourgoin BP (1990). *Mytilus edulis* shell as a bioindicator of lead pollution: considerations on bioavailability and variability. Marine Ecology Progress Series.

[CR7] Bulnheim HP, Gosling E (1988). Population genetic structure of mussels from the Baltic Sea. Helgoländer Meeresuntersuchungen.

[CR8] Carroll ML, Johnson BJ, Henkes GA, McMahon KW, Voronkov A, Ambrose WG, Denisenko SG (2009). Bivalves as indicators of environmental variation and potential anthropogenic impacts in the southern Barents Sea. Marine Pollution Bulletin.

[CR9] Cohen AL, Branch GM (1992). Environmentally controlled variation in the structure and mineralogy of *Patella granularis* shells from the coast of southern Africa: implications for palaeotemperature assessments. Palaeogeography, Palaeoclimatology, Palaeoecology.

[CR10] Cronin TM, Kamiya T, Dwyer GS, Belkin H, Vann CD, Schwede S, Wagner R (2005). Ecology and shell chemistry of *Loxoconcha matagordensis*. Palaeogeography, Palaeoclimatology, Palaeoecology.

[CR11] Dalbeck, P. C. (2008). *Crystallography, stable isotope and trace element analysis of Mytilus edulis shells in the context of ontogeny*. PhD thesis, University of Glasgow. http://theses.gla.ac.uk/1870/. Accessed 16 January 2017.

[CR12] Dettman DL, Flessa KW, Roopnarine PD, Schöne BR, Goodwin DH (2004). The use of oxygen isotope variation in shells of estuarine mollusks as a quantitative record of seasonal and annual Colorado River discharge. Geochimica et Cosmochimica Acta.

[CR13] Dissard D, Nehrke G, Reichart GJ, Bijma J (2010). Impact of seawater pCO_2_ on calcification and Mg/Ca and Sr/Ca ratios in benthic foraminifera calcite: results from culturing experiments with *Ammonia tepida*. Biogeosciences.

[CR14] Dodd JR (1963). Palaeoecological implications of shell mineralogy in two pelecypod species. Journal of Geology.

[CR15] Dodd JR (1965). Environmental control of strontium and magnesium in *Mytilus*. Geochimica et Cosmochimica Acta.

[CR16] Dodd JR (1966). Diagenetic stability of temperature-sensitive skeletal properties in *Mytilus* from the Pleistocene of California. Geological Society of America Bulletin.

[CR17] Dodd JR (1967). Magnesium and strontium in calcareous skeletons: a review. Journal of Paleontology.

[CR18] Dupont S, Dorey N, Thorndyke M (2010). What meta-analysis can tell us about vulnerability of marine biodiversity to ocean acidification?. Estuarine, Coastal and Shelf Science.

[CR19] Dziubińska, A., & Janas, U. (2007). Submerged objects—a nice place to live and develop. Succession of fouling communities in the Gulf of Gdańsk, Southern Baltic. *Oceanological and Hydrobiological Studies, 36*(4), 65–78.

[CR20] Eisma D, Mook WG, Das HA (1975). Shell characteristics, isotopic composition and trace-element contents of some euryhaline molluscs as indicators of salinity. Palaeogeography, Palaeoclimatology, Palaeoecology.

[CR21] Elderfield H, Ganssen G (2000). Past temperature and δ^18^O of surface ocean waters inferred from foraminiferal Mg/Ca ratios. Nature.

[CR22] Elliot M, Welsh K, Chilcott C, McCulloch M, Chappell J, Ayling B (2009). Profiles of trace elements and stable isotopes derived from giant long-lived *Tridacna gigas* bivalves: potential applications in paleoclimate studies. Palaeogeography, Palaeoclimatololgy, Palaeoecology.

[CR23] England, J. K. (2005). *Calcium carbonate biomineralisation in disparate systems – common mechanisms?* PhD thesis, University of Glasgow. http://theses.gla.ac.uk/4024/1/2005EnglandPhD.pdf. Accessed 16 January 2017.

[CR24] Findlay H, Tyrrell T, Bellerby R, Merico A, Skjelvan I (2007). Ecosystem modelling of the Norwegian Sea: investigating carbon and nutrients dynamics as a consequence of biological and physical processes. Biogeosciences Discussions.

[CR25] Franklin M, Morse J (1983). The interaction of manganese (II) with the surface of calcite in dilute solutions and seawater. Marine Chemistry.

[CR26] Freitas PS, Clarke LJ, Kennedy H, Richardson CA, Abrantes F (2006). Environmental and biological controls on elemental (Mg/Ca, Sr/Ca and Mn/Ca) ratios in shells of the king scallop *Pecten maximus*. Geochimica et Cosmochimica Acta.

[CR27] Fritioff Å, Kautsky L, Greger M (2005). Influence of temperature and salinity on heavy metal uptake by submersed plants. Environmental Pollution.

[CR28] Fuge R, Palmer TJ, Pearce NJG, Perkins WT (1993). Minor and trace element chemistry of modern shells: a laser ablation inductively coupled plasma spectrometry study. Applied Geochemistry.

[CR29] Gattuso JP, Hansson L, Gattuso JP, Hansson L (2011). Ocean acidification: background and history. Ocean acidification.

[CR30] Gillikin DP, Lorrain A, Navez J, Taylor JW, André L, Keppens E, Baeyens W (2005). Strong biological controls on Sr/Ca ratios in aragonitic marine bivalve shells. Geochemistry, Geophysics, Geosystems.

[CR31] Gillikin DP, Dehairs F, Baeyens W, Navez J, Lorrain A, Andre L (2005). Inter- and intra-annual variations of Pb/Ca ratios in clam shells (*Mercenaria mercenaria*): a record of anthropogenic lead pollution. Marine Pollution Bulletyn.

[CR32] Gillikin DP, Dehairs F, Lorrain A, Steenmans D, Baeyens W, André L (2006). Barium uptake into the shells of the common mussel (*Mytilus edulis*) and the potential for estuarine paleo-chemistry reconstruction. Geochimica et Cosmochimica Acta.

[CR33] Gofas, S. (2004). *Mytilus trossulus* Gould, 1850. In: *MolluscaBase* (2016). http://www.marinespecies.org/aphia.php?p=taxdetails&id=140482. Accessed 06 February 2017.

[CR34] Gray BE, Smith AM (2004). Mineralogical variation in shells of the blackfood abalone, *Haliotis iris* (Mollusca: Gastropoda: Haliotidae), in southern New Zealand. Pacific Science.

[CR35] Haderlie EC, Abbott DP, Morris RH, Abbott DP, Haderlie EC (1980). Bivalvia: the clams and allies. Intertidal invertebrates of California.

[CR36] Hahn S, Griesshaber E, Schmahl WW, Neuser RD, Ritter AC, Hoffmann R (2014). Exploring aberrant bivalve shell ultrastructure and geochemistry as proxies for past seawater acidification. Sedimentology.

[CR37] HELCOM (2004). The fourth Baltic Sea pollution load compilation (PLC-4). Baltic Sea Environmental Proceedings.

[CR38] Hendriks IE, Duarte CM, Olsen YS, Steckbauer A, Ramajo L, Moore (2015). Biological mechanisms supporting adaptation to ocean acidification in coastal ecosystems. Estuarine, Coastal and Shelf Science.

[CR39] Hiebenthal C, Philipp EER, Eisenhauer A, Wahl M (2012). Interactive effects of temperature and salinity on shell formation and general condition in Baltic Sea *Mytilus edulis* and *Arctica islandica*. Aquatic Biology.

[CR40] Hirao Y, Matsumoto A, Yamakawa H, Maeda M, Kimura K (1994). Lead behaviour in abalone shell. Geochimica et Cosmochimica Acta.

[CR41] Iglikowska A, Beldowski J, Chelchowski M, Chierici M, Kedra M, Przytarska (2017). Chemical composition of two mineralogically contrasting Arctic bivalves’ shells and their relationships to environmental variables. Marine Pollution Bulletin.

[CR42] Imai N, Terashima S, Itoh S, Ando A (1996). Compilation of analytical data on nine GSJ geochemical reference samples, “sedimentary rock series”. Geostandards Newsletter.

[CR43] Izumida, H., Yoshimura, T., Suzuki, A., Nakashima, R., Ishimura, T., Yasuhara, M., et al. (2011). Biological and water chemistry controls on Sr/Ca, Ba/Ca, Mg/Ca and δ^18^O profiles in freshwater pearl mussel *Hyriopsis* sp. *Palaeogeography, Palaeoclimatology, Palaeoecology, 309*, 298–308.

[CR44] Jackson AP, Vincent JFV, Turner RM (1988). The mechanical design of nacre. Proceedings of the Royal Society B: Biological Sciences.

[CR45] Kennedy WJ, Taylor JD, Hall A (1969). Environmental and biological controls on bivalve shell mineralogy. Biological Reviews.

[CR46] Klein, R. T., Lohmann, K. C., & Thayer, C. W. (1996a). Sr/Ca and ^13^C/^12^C ratios in skeletal calcite of *Mytilus trossulus*: covariation with metabolic rate, salinity, and carbon isotopic composition of seawater. *Geochimica et Cosmochimica Acta, 60*, 4207–4221.

[CR47] Klein RT, Lohmann KC, Thayer CW (1996). Bivalve skeletons record sea-surface temperatures and ^18^O via Mg/Ca and ^18^O/^16^O ratios. Geology.

[CR48] Krzeminska M, Kuklinski P, Najorka J, Iglikowska A (2016). Skeletal mineralogy patterns of Antarctic Bryozoa. Journal of Geology.

[CR49] Kuklinski P, Taylor PD (2009). Mineralogy of Arctic bryozoan skeletons in a global context. Facies.

[CR50] Langston WJ, Bebianno MJ, Burt GR, Langston WJ, Bebianno MJ (1998). Metal handling strategies in molluscs. Metal metabolism in aquatic environments.

[CR51] Lauringson V, Kotta J, Orav-Kotta H, Kaljurand K (2013). Diet of mussels *Mytilus trossulus* and *Dreissena polymorpha* in a brackish nontidal environment. Marine Ecology.

[CR52] Lazareth CE, Vander Putten E, Andre L, Dehairs F (2003). High-resolution trace element profiles in shells of the mangrove bivalve *Isognomon ephippium*: a record of environmental spatio-temporal variations?. Estuarine Coastal Shelf Science.

[CR53] Lear C, Rosenthal Y, Slowey N (2002). Benthic foraminiferal Mg/Ca paleothermometry: a revised core-top calibration. Geochimica et Cosmochimica Acta.

[CR54] Lee J, Morse JW (2010). Influences of alkalinity and pCO_2_ on CaCO_3_ nucleation from estimated Cretaceous composition seawater representative of “calcite seas”. Geology.

[CR55] Lee BG, Wallace WG, Luoma SN (1998). Uptake and loss kinetics of Cd, Cr and Zn in the bivalves *Potamocorbula amurensis* and *Macoma balthica*: effects of size and salinity. Marine Ecology Progress Series.

[CR56] Lindström M (2001). Urban land use influences on heavy metal fluxes and surface sediment concentrations of small lakes. Water, Air and Soil Pollution.

[CR57] Lorens R, Bender M (1980). The impact of solution chemistry on *Mytilus edulis* calcite and aragonite. Geochimica et Cosmochimica Acta.

[CR58] Lorrain A, Gillikin D, Paulet YM, Chavaud L, Lemercier A, Navez J, Andre L (2005). Strong kinetic effects on Sr/Ca ratios in the calcitic bivalve *Pecten maximus*. Geology.

[CR59] Lowenstam HA (1954). Factors affecting the aragonite:calcite ratios in carbonate-secreting marine organisms. Journal of Geology.

[CR60] Lowenstam HA (1981). Minerals formed by organisms. Science.

[CR61] Lowenstam HA, Weiner S (1989). On biomineralization.

[CR62] Malone P, Dodd J (1967). Temperature and salinity effects on calcification rate in *Mytilus edulis* and its paleoecological implications. Limnology Oceanography.

[CR63] Mann S (2001). Biomineralization: principles and concepts in bioinorganic materials chemistry.

[CR64] Markich SJ, Jeffree RA, Burke PT (2002). Freshwater bivalve shells as archival indicators of metal pollution from a copper-uranium mine in tropical northern Australia. Environmental Science & Technology.

[CR65] Morse JW, Andersson AJ, MacKenzie F (2006). Initial responses of carbonate-rich shelf sediments to rising atmospheric pCO_2_ and ‘ocean acidification’: role of high Mg-calcite. Geochimica et Cosmochimica Acta.

[CR66] Newman MC, Heagler MG, Newman MC, McIntosh AW (1991). Allometry of metal bioaccumulation and toxicity. Metal ecotoxicology: concepts and applications.

[CR67] Paerl HW, Bales JD, Ausley LW, Buzzelli CP, Crowder LB, Eby LA (2001). Ecosystem impacts of three sequential hurricanes (Dennis, Floyd, and Irene) on the United States’ largest lagoonal estuary, Pamlico Sound, NC. Proceedings of the National Academy of Sciences of the United States of America.

[CR68] Palacios R, Orensanz JM, Armstrong DA (1994). Seasonal and life-long variation of Sr/Ca ratio in shells of *Mya arenaria* from Grays Harbor (Washington)—an ancillary criterion in demographic studies. Estuarine Coastal and Shelf Science.

[CR69] Palmer AR (1992). Calcification in marine molluscs: how costly is it?. Proceedings of the National Academy of Sciences.

[CR70] Pierścieniak K, Grzymała J, Wołowicz M (2010). Differences in reproduction and condition of *Macoma balthica* and *Mytilus trossulus* in the Gulf of Gdansk (southern Baltic Sea) under anthropogenic influences. Oceanological and Hydrobiological Studies.

[CR71] Pitts LC, Wallace GT (1994). Lead deposition in the shell of the bivalve, *Mya arenaria*: an indicator of dissolved lead in seawater. Estuarine, Coastal and Shelf Science.

[CR72] Poulain C, Gillikin DP, Thébault J, Munaron JM, Bohn M, Robert R (2015). An evaluation of Mg/Ca, Sr/Ca, and Ba/Ca ratios as environmental proxies in aragonite bivalve shells. Chemical Geology.

[CR73] Protasowicki M, Dural M, Jaremek J (2008). Trace metals in the shells of blue mussels (*Mytilus edulis*) from the Poland coast of Baltic Sea. Environmental Monitoring and Assessment.

[CR74] Puente X, Villares R, Carral E, Carballeira A (1996). Nacreous shell of *Mytilus galloprovincialis* as a biomonitor of heavy metal pollution in Galiza (NW Spain). Science of the Total Environment.

[CR75] Putten EV, Dehairs F, Keppens E, Baeyens W (2000). High resolution distribution of trace elements in the calcite shell layer of modern *Mytilus edulis*: environmental and biological controls. Geochimica et Cosmochimica Acta.

[CR76] Rainbow PS, Furness RW, Rainbow PS (1990). Heavy metals in marine invertebrates. Heavy metals in the marine environment.

[CR77] Rainbow PS, Amiard-Triquet C, Amiard JC, Smith BD, Best SL, Nassiri Y, Langston WJ (1999). Trace metal uptake rates in crustaceans (amphipods and crabs) from coastal sites in NW Europe differentially enriched with trace metals. Marine Ecology Progress Series.

[CR78] Rainbow, P. S., Wolowicz, M., Fialkowski, W., Smith, B. D., & Sokolowski, A. (2000). Biomonitoring of trace metals in the Gulf of Gdansk, using mussels (*Mytilus trossulus*) and barnacles (*Balanus improvisus*). *Water Research, 34*(6), 1823–1829.

[CR79] Rainbow PS, Fialkowski W, Sokolowski A, Smith BD, Wolowicz M (2004). Geographical and seasonal variation of trace metal bioavailabilities in the Gulf of Gdansk, Baltic Sea using mussels (*Mytilus trossulus*) and barnacles (*Balanus improvisus*) as biomonitors. Marine Biology.

[CR80] Ramajo L, Rodriguez-Navarro A, Duarte CM, Lardies MA, Lagos NA (2015). Shifts in shell mineralogy and metabolism of *Concholepas concholepas* juveniles along the Chilean coast. Marine Freshwater Research.

[CR81] Reiss D, Rihm B, Thöni C, Faller M (2004). Mapping stock at risk and release of zinc and copper in Switzerland—dose response functions for runoff rates derived from corrosion rate data. Water, Air and Soil Pollution.

[CR82] Richardson C (2001). Molluscs as archives of environmental change. Oceanography and Marine Biology.

[CR83] Ricketts EF, Calvin J (1971). Between Pacific tides.

[CR84] Riisgård HU, Lüskow F, Pleissner D, Lundgreen K, López MAP (2013). Effect of salinity on filtration rates of mussels *Mytilus edulis* with special emphasis on dwarfed mussels from the low saline central Baltic Sea. Helgoland Marine Research.

[CR85] Riisgård HU, Mulot M, Merino L, Pleissner D (2014). Effect of salinity-changing rates on filtration activity of mussels from two sites within the Baltic Mytilus hybrid zone: the brackish Great Belt (Denmark) and the low saline central Baltic Sea. Open Journal of Marine Science.

[CR86] Rosenberg GD, Rhoads DC, Lutz RA (1980). An ontogenetic approach to the environmental significance of bivalve shell chemistry. Skeletal growth of aquatic organisms: biological records of environmental change.

[CR87] Salisbury J, Green M, Hunt C, Campbell J (2008). Coastal acidification by rivers: a threat to shellfish?. EOS, Transactions, American Geophysical Union.

[CR88] Schöne BR, Zhang Z, Radermacher P, Thébault J, Jacob DE, Nunn EV (2011). Sr/Ca and Mg/Ca ratios of ontogenetically old, long-lived bivalve shells (*Arctica islandica*) and their function as palaeotemperature proxies. Palaeogeography, Palaeoclimatology, Palaeoecology.

[CR89] Smith SV, Buddemeier RW, Redalje RC, Houck JE (1979). Strontium–calcium thermometry in coral skeletons. Science.

[CR90] Staniszewska M, Nehring I, Zgrundo A (2015). The role of phytoplankton composition, biomass and cell volume in accumulation and transfer of endocrine disrupting compounds in the southern Baltic Sea (The Gulf of Gdansk). Environmental Pollution.

[CR91] State Environmental Monitoring (2014). Wstępna ocena stanu środowiska wód morskich polskiej strefy Morza Bałtyckiego. *European Commission Report* (pp. 462). Warszawa. https://www.mos.gov.pl/g2/big/2014_04/9cbf6a1fad8785948a0931cadfdf7ce9.pdf/. Accessed.

[CR92] Stecher HA, Krantz DE, Lord CJ, Luther GW, Bock KW (1996). Profiles of strontium and barium in *Mercenaria mercenaria* and *Spisula solidissima* shells. Geochimica et Cosmochimica Acta.

[CR93] Strasser CA, Mullineaux LS, Walther BD (2008). Growth rate and age effects on *Mya arenaria* shell chemistry: implications for biogeochemical studies. Journal of Experimental Marine Biology and Ecology.

[CR94] Stumpp M, Dupont S, Thorndyke MC, Melzner F (2011). CO_2_ induced seawater acidification impacts sea urchin larval development II: gene expression patterns in pluteus larvae. Comparative Biochemistry and Physiology-Part A: Molecular & Integrative Physiology.

[CR95] Swart PK, Grottoli A (2003). Proxy indicators of climate in coral skeletons: a perspective. Coral Reefs.

[CR96] Szefer, P., & Szefer, K. (1990). Metals in molluscs and associated bottom sediments of the southern Baltic. *Helgoländer Meeresuntersuchungen, 44*, 411–424.

[CR97] Szefer P, Frelek K, Szefer K, Lee CB, Kim BS, Warzocha J (2002). Distribution and relationships of trace metals in soft tissue, byssus and shells of *Mytilus edulis trossulus* from the southern Baltic. Environmental Pollution.

[CR98] Taylor JD, Layman MA (1972). The mechanical properties of bivalve (Mollusca) shell structure. Palaeontology.

[CR99] Thébault J, Chauvaud L, L’Helguen S, Clavier J, Barats A, Jacquet S (2009). Barium and molybdenum records in bivalve shells: geochemical proxies for phytoplankton dynamics in coastal environments?. Limnology and Oceanography.

[CR100] Tyrrell T, Schneider B, Charalampopoulou A, Riebesell U (2007). Coccolithophores and calcite saturation state in the Baltic and Black Seas. Biogeosciences Discussions.

[CR101] Uścinowicz S (2011). Geochemia osadów powierzchniowych Morza Bałtyckiego.

[CR102] Väinölä R, Strelkov P (2011). *Mytilus trossulus* in northern Europe. Marine Biology.

[CR103] Wang WX, Fisher NS (1997). Modeling the influence of body size on trace element accumulation in the mussel *Mytilus edulis*. Marine Ecology Progress Series.

[CR104] Watabe N, Wilbur KM (1960). Influence of the organic matrix on crystal type in molluscs. Nature.

[CR105] Wojcik D, Normant M, Dmochowska B, Fowler A (2015). Impact of Chinese mitten crab *Eriocheir sinensis* on blue mussel *Mytilus edulis trossulus* laboratory studies of claw strength, handling behavior, consumption rate, and size selective predation. Oceanologia.

[CR106] Wong WH, Levinton JS (2004). Culture of the blue mussel *Mytilus edulis* (Linnaeus, 1758) fed both phytoplankton and zooplankton: a microcosm experiment. Aquaculture Research.

[CR107] Wright DA (1995). Trace metal and major ion interactions in aquatic animals. Marine Pollution Bulletin.

[CR108] Wright DA, Zamuda CD (1987). Copper accumulation by two bivalves: salinity effect is independent of cupric ion activity. Marine Environmental Research.

[CR109] Youssef M, El-Sorogy A, El-Sabrouty M, Al-Otaibi N (2016). Invertebrate shells as pollution bio-indicators, Gebel El-Zeit area, Gulf of Suez, Egypt. Indian Journal of Geo-Marine Sciences.

[CR110] Zhang C, Zhang R (2006). Matrix proteins in the outer shells of molluscs. Marine Biotechnology.

[CR111] Zhong S, Mucci A (1989). Calcite and aragonite precipitation from seawater solutions of various salinities: precipitation rates and overgrowth compositions. Chemical Geology.

[CR112] Zhuravlev AY, Wood RA (2009). Controls on carbonate skeletal mineralogy: global CO_2_ evolution and mass extinctions. Geology.

[CR113] Zuschin M, Stachowitsch M, Stanton RJ (2003). Patterns and processes of shell fragmentation in modern and ancient marine environments. Earth-Science Reviews.

[CR114] Zuykov M, Pelletier E, Harper DAT (2013). Bivalve mollusks in metal pollution studies: from bioaccumulation to biomonitoring. Chemosphere.

